# Adherence to dietary and physical activity guidelines and influencing factors among pregnant women: a cross-sectional study in China

**DOI:** 10.3389/fnut.2026.1775351

**Published:** 2026-04-16

**Authors:** Wanling Hou, Lun Wang, Yinying Huang, Yumin Lin, Yalan Chen, Hongyu He, Lili Zhao

**Affiliations:** 1 Women and Children’s Hospital, School of Medicine, Xiamen University, Xiamen, China; 2 Zhongshan Hospital, Xiamen University, Xiamen, China; 3 Fujian Health College, Fuzhou, China

**Keywords:** adherence, diet, influencing factors, physical activity, pregnant woman

## Abstract

**Introduction:**

The diet and physical activity of pregnant women are crucial for maternal and fetal health. Many countries have developed guidelines. Research shows higher adherence to dietary guidelines is associated with reduced risks of complications. However, pregnant women’s adherence to these guidelines remains low. Accurate assessment of current adherence levels is essential to characterize behavioral patterns, identify high-risk groups, and provide evidence for the development of targeted interventions aimed at supporting maternal–fetal health. The aim of this study was to investigate the adherence of pregnant women to dietary and physical activity guidelines and analyze factors associated with adherence, thereby providing a foundation for developing intervention strategies in clinical practice.

**Methods:**

Pregnant women who received routine prenatal care at the Department of Obstetrics, Women and Children’s Hospital, School of Medicine, Xiamen University, from January to June 2025 were enrolled as study participants. A cross-sectional survey was conducted using general information, Pregnant Women’s Dietary Guideline Compliance Index questionnaire, and physical activity guideline recommendations to analyze influencing factors.

**Results:**

A total of 327 valid questionnaires were returned. The mean dietary and physical activity adherence score was 59.78 (SD = 16.04). Univariate analysis showed significant differences in adherence by ethnicity, stage of gestation, exercise habits during pregnancy, education level, occupation, personal monthly income, and family monthly income (all *P* < 0.05). Multiple linear regression analysis showed that stage of gestation and exercise habits during pregnancy remained significantly associated with adherence to guideline recommendations for diet and physical activity after adjusting for confounders (both *P* < 0.05).

**Conclusion:**

Pregnant women exhibited a moderate level of adherence to dietary and physical activity guidelines. These findings suggest that healthcare providers may consider developing personalized guidance plans based on individual profiles, though longitudinal intervention studies are needed to determine their impact on pregnancy outcomes.

## 1 Introduction

Adequate nutrition and appropriate physical activity constitute vital elements of prenatal care. Both are directly linked to maternal-fetal health. Balanced dietary patterns, combined with regular exercise, help control gestational weight gain, reduce cesarean delivery rates, and lower neonatal complication risks (1). Consequently, numerous countries have developed guidelines for maternal diet and physical activity (2–4). These aim to standardize health behaviors and promote healthy lifestyles during pregnancy. In China, Ding et al. conducted a prospective cohort study in Wuhan to investigate the impact of diet and lifestyle on maternal and offspring health (5). Their results indicated that higher adherence to dietary guidelines was associated with reduced risks of gestational hypertension and diabetes mellitus, consistent with other studies (6, 7). Additionally, a national birth cohort study demonstrated that improved maternal diet quality benefits neurodevelopment in children aged 1–3.5 years (8). Despite these established benefits, compliance with recommendations remains poor among pregnant women (9).

A study assessing dietary quality during early pregnancy (gestational days 77–161) found a mean Healthy Eating Index (HEI) score of 60.63 (SD = 2.91), reflecting moderate dietary quality (10). The HEI ranges from 0 to 100, with higher scores reflecting better adherence to dietary guidelines and superior dietary quality. Another study, which evaluated the dietary intake consistency with guidelines among 10,038 singleton pregnant women, revealed that the average total HEI scores were 57–59 for Black women, 61–66 for White women, 61–63 for Hispanic women, and 66–69 for Asian women (11). Additionally, a study conducted in China showed that the dietary quality score of pregnant women was 74.1 (SD = 7.5), which still failed to meet the guideline standards (5). Despite the fact that most women pay more attention to dietary quality during pregnancy, their dietary adjustments are often limited to avoiding alcohol, caffeine, and foods with potential safety concerns, rather than making comprehensive improvements to overall dietary quality (12, 13).

National dietary guidelines are developed to address population-level nutritional requirements. They incorporate broad food categories to ensure generalizability across settings. However, China encompasses vast geographic regions with heterogeneous dietary cultures. Pregnant women often make food choices based on local eating customs and resource availability. Such regional dietary habits may differ from guideline recommendations.

Beyond dietary behaviors, compliance with physical activity recommendations is equally concerning worldwide. An Australian study assessed weekly physical activity in 780 pregnant women and reported that only 7% met the national guidelines (14). Similarly, a Spanish study found that merely 22% of pregnant women achieved recommended activity levels (15). Although guidelines vary in their specifics (frequency, duration, intensity, and type), all recommend moderate-intensity exercise for healthy pregnant women without contraindications (16, 17). However, studies consistently demonstrate that physical activity levels remain suboptimal in this population (18–20).

Therefore, this study aims to assess current maternal diet and physical activity against guidelines to characterize adherence patterns in this population. Understanding these patterns may inform future interventions aimed at enhancing awareness and supporting guideline adherence. While adequate nutrition and physical activity are associated with improved maternal-fetal outcomes, the present cross-sectional study focuses on describing current compliance levels rather than establishing causal relationships or intervention efficacy.

## 2 Materials and methods

### 2.1 Study design and setting

The study employed a cross-sectional design and adhered to the Strengthening the Reporting of Observational Studies in Epidemiology (STROBE) guidelines for cross-sectional research.

### 2.2 Sampling and sample size

Participants were recruited using convenience sampling. Eligible women were receiving regular antenatal care at Women’s and Children’s Hospital, Xiamen University School of Medicine between October 2024 and January 2025. Inclusion criteria for the study included pregnant women who met the following criteria: (I) aged 20 years old and above; (II) singleton pregnancy; (III) normal communication and reading ability; (IV) provided informed consent. Pregnant women were excluded if they had: (I) Pregnancy complications or comorbidities (e.g., placenta previa, placental abruption, fetal growth restriction, threatened preterm labor); (II) Eating disorders or hyperthyroidism; (III) Disabilities; (IV) Absolute contraindications to exercise during pregnancy (e.g., severe cardiovascular disease, respiratory disease, uncontrolled type 1 diabetes, cervical insufficiency, after cervical cerclage). According to the Kendall sample size method, the appropriate sample size is 5–10 times the number of variables. This study had 13 variables. Twenty percent of the calculated sample size was added to account for potential attrition, resulting in a minimum required sample size of 156. A total of 327 cases were collected. This study was approved by the Ethics Review Committee of Women’s and Children’s Hospital, School of Medicine, Xiamen University (No. KY-2020-106).

### 2.3 Data collection instruments

Pregnant women completed a basic information questionnaire, which included such as ethnicity, age, residence, height, pre-pregnancy weight, pregnancy weight, occupation, educational level, monthly income, family monthly income, gestational week, parity, and delivery history.

Pregnant women’s compliance with the dietary guidelines was assessed using the Pregnant Women’s Dietary Guideline Compliance Index (21). This validated index demonstrates strong alignment with the Chinese Balanced Dietary Pagoda for women during preconception and pregnancy. It evaluates compliance with key dietary recommendations. The index also facilitates nutritional counseling during pregnancy. It consists of 13 scoring criteria, totaling 100 points, and is used to assess the dietary intake of pregnant women in early, mid, and late pregnancy.

Guidelines from the American College of Obstetricians and Gynecologists (22) and the Chinese Society of Perinatal Medicine, China Medical Association (23) advise 150 min of weekly moderate-intensity exercise for pregnant women without contraindications. This can be achieved through 30-min sessions on 5 days, or accumulated over three or more days. Accordingly, two items from the questionnaire developed by Dubasi et al. (24) were used. This questionnaire has demonstrated good internal consistency (Cronbach’s α = 0.94). The two items capture the two core components of physical activity guidelines: frequency and duration. Frequency was assessed by the question: “How many days do you exercise in a week?” Response options were: 1 = never; 2 = 1–2 days/week; 3 = 3–4 days/week; 4 = 5–6 days/week; 5 = daily. Duration was assessed by the question: “How much time do you exercise for each session?” Response options were: 1 ≤ 10 min; 2 = 10–20 min; 3 = 20–30 min; 4 = 30–40 min; 5 ≥ 40 min. Each item was scored on a 1–5 scale. The total score was calculated by summing the two items (range: 2–10). For this study, the original format was modified. The two items were changed to open-ended questions. Actual exercise days and minutes were reported by participants. These responses were subsequently categorized into the corresponding options for score calculation.

### 2.4 Data collection

Data were collected through on-site questionnaires. After obtaining consent from the participants, the questionnaires were distributed on-site. Once the participants completed their check-ups, they filled out the questionnaires anonymously. Before filling out the questionnaires, the investigators ensured that the study’s purpose, methods, and detailed instructions were fully explained. The participants completed the questionnaires independently. The investigators then collected and checked each questionnaire to ensure the authenticity and validity of the survey results.

### 2.5 Data analysis

The normality of variable distributions was assessed. The total adherence score for diet and physical activity was a continuous variable (skewness = −0.267, kurtosis = −0.119) and followed a normal distribution. Measurement data are presented as mean ± standard deviation (SD). Differences between two groups were assessed using independent samples *t*-tests. Categorical data are reported as percentages (%) and were evaluated using chi-square (χ^2^) tests. Multiple linear regression was performed with the total adherence score as the dependent variable and factors significant in univariate analysis as independent variables. A two-sided *P*-value < 0.05 was considered statistically significant for all analyses. Variable selection for multivariable regression followed a three-step procedure. First, potential predictors were identified based on existing literature regarding determinants of dietary and physical activity adherence in pregnant populations (3, 15, 25, 26). Second, variables showing significant associations (*P* < 0.05) in univariate analyses were considered for inclusion. Third, clinically relevant factors (e.g., parity, pre-pregnancy BMI) were entered into the model regardless of univariate significance. All analyses were performed using SPSS Statistics version 26.0 (IBM Corp., Armonk, NY, USA).

## 3 Results

### 3.1 Demographic characteristics of the participants

A total of 327 cases were collected. The age range was 20–41 years, with a mean age of 30.23 ± 4.03 years. The average height was 160.50 ± 5.43 cm, and the average pre-pregnancy weight was 54.88 ± 8.50 kg. The mean pre-pregnancy BMI was 21.32 ± 3.31 kg/m^2^. Among the participants, 262 (80.12%) pregnant women lived in urban areas, while 65 (19.88%) lived in rural areas. In terms of ethnicity, 97.41% were Han Chinese, and 2.59% were ethnic minorities. Regarding parity, the majority of pregnant women were primiparous (214, 65.4%), while 113 (34.6%) were multiparous. The mean number of pregnancies was 1.56 ± 0.98. Of the participants, 24 (7.34%) had a postgraduate degree, 171 (52.29%) had a bachelor’s degree, 91 (27.83%) had a college degree, and 41 (12.54%) had a high school education or below.

### 3.2 Pregnant women’s adherence to dietary and physical guidelines

The overall mean adherence score for diet and physical activity among pregnant women was 59.78 (SD = 16.04). Specifically, the mean dietary adherence score was 52.72 (SD = 15.63), and the mean physical activity adherence score was 7.06 (SD = 1.53). See Table 1 for details.

**TABLE 1:** The total score of adherence for diet and physical activity (*n* = 327).

Variable	Number of items	Total scores	Average total scores
Diet	13	100	52.72 ± 15.63
Physical activity	2	10	7.06 ± 1.53
Total	15	110	59.78 ± 16.04

### 3.3 Factors influencing adherence to dietary and physical activity guidelines among pregnant women

Univariate analysis of adherence scores for diet and physical activity showed significant differences by ethnicity, stage of gestation, exercise habits during pregnancy, education level, occupation, monthly income and family monthly income (all *P* < 0.05), as shown in Table 2.

**TABLE 2:** Univariate analysis of adherence to diet and physical activity in pregnant women (*n* = 327).

Variable	*n* (%)	Average total scores	*F/t*	*P*
Age			−1.496	0.136
20∼34	277 (84.71%)	59.22 ± 16.02		
≥35	50 (15.29%)	62.9 ± 15.95		
Residence			1.473	0.144
Urban areas	262 (80.12%)	60.52 ± 15.23		
Rural areas	65 (19.88%)	56.81 ± 18.80		
Ethnicity			−2.007	0.046
Han Chinese	312 (95.41%)	59.39 ± 16.16		
Ethnic minorities	15 (4.59%)	67.87 ± 10.88		
Delivery history			−0.199	0.842
No	214 (65.44%)	59.65 ± 15.37		
Yes	113 (34.56%)	60.03 ± 17.31		
Parity			0.561	0.571
1	196 (59.94%)	59.76 ± 15.40		
2	96 (29.36%)	58.93 ± 16.33		
≥3	35 (10.7%)	62.29 ± 18.81		
Stage of gestation			−4.638	<0.001
The first trimester	109 (33.33%)	59.98 ± 16.46		
The second and third trimesters of pregnancy	218 (66.67%)	67.18 ± 12.86		
Exercise habits during pregnancy			−4.489	<0.001
No	156 (47.71%)	55.73 ± 16.32		
Yes	171 (52.29%)	63.48 ± 14.90		
Working or not during pregnancy			−0.985	0.325
No	30 (9.17%)	57.03 ± 20.84		
Yes	297 (90.83%)	60.06 ± 15.49		
Pre-pregnancy BMI			1.717	0.163
Skinny	51 (15.60%)	56.74 ± 16.83		
Normal	220 (67.28%)	61.11 ± 15.76		
Overweight	42 (12.84%)	56.47 ± 16.86		
Fat	14 (4.28%)	59.93 ± 13.54		
Education level			6.185	<0.001
High school education or below	41 (12.54%)	53.75 ± 16.47		
College degree	91 (27.83%)	56.29 ± 15.02		
Bachelor’s degree	171 (52.29%)	62.20 ± 16.25		
Postgraduate degree	24 (7.34%)	66.08 ± 11.98		
Occupation			3.205	0.023
Personnel of enterprises or institutions	34 (10.4%)	63.59 ± 14.99		
Professional technicians	57 (10.4%)	62.44 ± 15.53		
Commercial and service personnel	65 (10.4%)	62.24 ± 13.43		
Others	171 (52.29%)	57.20 ± 16.98		
Monthly income			14.563	<0.001
<3000 RMB	38 (11.62%)	52.79 ± 17.38		
3000∼7000 RMB	138 (42.2%)	55.59 ± 16.17		
7000∼10000 RMB	90 (27.52%)	63.28 ± 12.67		
≥10000 RMB	61 (18.65%)	68.48 ± 14.49		
Family monthly income			10.992	<0.001
<5000 RMB	16 (4.89%)	48.69 ± 19.24		
5000∼10000 RMB	82 (25.08%)	53.63 ± 15.36		
10000∼30000 RMB	177 (54.13%)	61.83 ± 15.02		
≥30000 RMB	52 (15.9%)	65.92 ± 15.20		

Multiple linear regression analysis was performed using the total adherence score for diet and physical activity as the outcome variable. Predictor variables comprised factors significant at *P* < 0.05 in univariate analysis, together with clinically important variables (parity, pre-pregnancy BMI) identified from prior research. After adjusting for confounders, Stage of gestation and exercise habits during pregnancy remained significantly associated with adherence to guideline recommendations (*P* < 0.05), accounting for 16.1% of the total variance (Table 3).

**TABLE 3:** Multivariate linear regression analysis of adherence to diet and physical activity in pregnant women (*n* = 327).

Variables	*B*	SE	β	*t*	*p*	95% CI
(Intercept)	51.254	6.110	–	8.224	<0.001	38.230∼62.277
Ethnicity						
Ethnic minorities	Ref.					
Han Chinese	−6.006	4.034	−0.078	−1.489	0.138	−13.944∼1.933
Parity						
1	Ref.					
2	−1.058	1.924	−0.030	−0.550	0.583	−4.844∼2.727
≥3	2.264	2.804	0.044	0.807	0.420	−3.253∼7.781
Stage of gestation						
The first trimester	Ref.					
The second and third trimesters of pregnancy	7.286	1.853	0.109	4.618	<0.001	3.514∼14.604
Exercise habits during pregnancy						
No	Ref.					
Yes	7.509	1.668	0.234	4.502	<0.001	4.227∼10.791
Pre-pregnancy BMI						
Skinny	Ref.					
Normal	1.021	2.394	0.030	0.426	0.670	−3.690∼5.7332
Overweight	−0.918	3.154	−0.019	−0.291	0.771	−7.124∼5.288
Fat	2.619	4.531	0.033	0.578	0.564	−6.298∼11.535
Education level						
High school education or below	Ref.					
College degree	−0.344	2.982	−0.010	−0.116	0.908	−6.211∼5.522
Bachelor’s degree	2.927	3.005	0.091	0.974	0.331	−2.985∼8.840
Postgraduate degree	1.941	4.470	0.032	0.434	0.665	−6.856∼10.737
Occupation						
Others	Ref.					
Personnel of enterprises or institutions	2.846	2.902	0.054	0.981	0.327	−2.863∼8.556
Professional technicians	1.307	2.408	0.031	0.543	0.588	−3.431∼6.044
Commercial and service personnel	1.957	2.297	0.049	0.852	0.395	−2.562∼6.476
Monthly income						
<3000 RMB	Ref.					
3000∼7000 RMB	−1.106	3.154	−0.034	−0.351	0.726	−7.312∼5.101
7000∼10000 RMB	4.283	3.745	0.119	1.144	0.254	−3.086∼11.652
≥10000 RMB	6.611	4.187	0.161	1.579	0.115	−1.628∼14.850
Family monthly income						
<5000 RMB	Ref.					
5000∼10000 RMB	3.125	4.389	0.085	0.712	0.477	−5.512∼11.762
10000∼30000 RMB	6.350	4.557	0.198	1.393	0.164	−2.617∼15.318
≥30000 RMB	8.868	5.064	0.202	1.751	0.081	−1.096∼18.832

Ref., Reference; *R* = 0.461, *R*^2^ = 0.212, adjusted *R*^2^ = 0.161, *F* = 4.126, *P* < 0.001.

## 4 Discussion

Dietary nutrition and physical activity during pregnancy remain a critical public health priority. These factors are strongly associated with maternal and offspring health outcomes (27). Our study revealed a mean total adherence score for diet and physical activity of 59.78 (SD = 16.04) on a 110-point scale. This corresponds to 54.24% (SD = 14.58%) when converted to a 100-point percentage scale. This indicates moderate compliance among pregnant women. These findings align with global observations of suboptimal adherence. A systematic review by Caut et al. (25) reported that pregnant women across 10 countries consistently failed to meet minimum dietary guidelines. A study conducted in the United States found that only 3% of pregnant women met the 2020–2025 Dietary Guidelines for Americans (26). In Spain, objective accelerometer measurements indicated that 22% of pregnant women adhered to physical activity guidelines (15). Similarly, more than half of pregnant women in an Australian cohort failed to meet relevant recommendations (28). Low adherence has also been observed in studies of Chinese populations (29). The consistency of these findings across diverse healthcare systems and assessment methods suggests systemic barriers to guideline implementation, potentially reflecting inadequate professional guidance and limited health awareness (30). Educational interventions have demonstrated feasibility and acceptability for modifying maternal lifestyle behaviors (31). Therefore, future health promotion strategies might prioritize enhancing professional guidance and health education to facilitate guideline adherence. Healthcare providers could consider personalized counseling approaches, particularly for women with low baseline adherence; however, given the cross-sectional nature of the current study, whether such interventions improve pregnancy outcomes requires verification through prospective or randomized studies.

The results of this study show that pregnant women in the second and third trimesters were more likely to report adherence to dietary and physical activity recommendations than those in the first trimester. Potential explanations for this association include the increased nutritional demands of fetal development as pregnancy progresses, particularly in the second and third trimesters, which may coincide with greater attention to dietary intake to meet these needs. Additionally, the subsiding of early pregnancy symptoms (e.g., morning sickness) and more noticeable weight gain in later trimesters may be associated with increased awareness of dietary habits and physical activity for weight management. These findings are consistent with previous cross-sectional studies showing higher guideline adherence in later trimesters (21, 32). Healthcare providers may consider prioritizing dietary and physical activity counseling for first-trimester women; however, longitudinal intervention studies are needed to determine whether such targeted support improves adherence and maternal-infant health outcomes.

Exercise habits, a modifiable factor associated with favorable pregnancy outcomes (33), were found to correlate with higher guideline adherence in our cross-sectional sample. While WHO recommends 150 min of weekly physical activity during pregnancy (34), only 37.0% of participants met this criterion, consistent with prior observations (35). This low prevalence is particularly concerning given documented declines in physical activity during gestation (36). Although guideline adherence has been associated with improved quality of life (37), the cross-sectional design precludes causal inference. Future prospective studies should evaluate whether targeted exercise counseling, particularly for women with low baseline activity, improves adherence and pregnancy outcomes.

While our findings suggest that gestational stage and exercise habits represent modifiable correlates of guideline adherence, we emphasize that causal efficacy remains unproven. Our data indicate that first-trimester women and those without established exercise routines represent high-priority subgroups for intervention development. Future randomized controlled trials should test whether trimester-specific counseling effectively improves adherence compared with standard care. However, several methodological limitations warrant consideration. Our regression model explained only 16.1% of the variance in adherence, indicating substantial unexplained heterogeneity. This limited explanatory power likely reflects the multifactorial nature of health behaviors and unmeasured factors (e.g., social support, psychological stress, food environment accessibility). Parity and pre-pregnancy BMI did not substantially increase explained variance, suggesting these clinical characteristics may exert indirect effects through behavioral mediators. Therefore, the identified associations should be interpreted as partial relationships rather than comprehensive causal explanations. Future research should adopt an integrated biopsychosocial framework to identify additional determinants and improve predictive models.

### 4.1 Limitations

Several limitations of the present study warrant consideration. First, given the cross-sectional nature of this study, we cannot establish the temporal sequence between influencing factors and adherence. Future prospective studies are essential to delineate temporal trajectories and assess whether baseline adherence predicts subsequent maternal-fetal outcomes (e.g., gestational diabetes, birth weight), thereby strengthening causal inference beyond the associations observed in this study. Second, the study population was recruited using convenience sampling, which may compromise the representativeness of the sample and limit the generalizability of the findings to the broader population. The sample was predominantly urban and highly educated, potentially limiting external validity and generalizability to rural or lower-socioeconomic-status populations. Moreover, this urban-centric recruitment may mask geographical disparities in environmental exposures, nutritional status, and antenatal care quality between settings (38). To enhance representativeness, future research should implement stratified random sampling across diverse socioeconomic and geographical contexts. Third, this study focused exclusively on pregnant women in China, which restricts the extrapolation of our findings to other populations, such as those from different countries. Finally, data were collected via self-administered questionnaires, requiring participants to recall their dietary intake and physical activity over the past month. This approach may introduce recall bias. Furthermore, the self-reported physical activity assessment was simplified. It captured frequency and duration. However, it did not assess intensity or activity type. These components are specified in current guidelines. Consequently, measurement validity may be limited. Comparability with objective measures, such as accelerometers, is also reduced. Future studies should employ objective monitoring devices to enhance precision.

## 5 Conclusion

In summary, adherence to diet and physical activity guidelines among pregnant women was suboptimal, highlighting the need for individualized counseling. Gestational week and exercise habits during pregnancy were significantly associated with guideline adherence. While these findings suggest that clinical medical staff may consider providing personalized, targeted guidance based on individual dietary and physical activity profiles to potentially enhance adherence, the cross-sectional design precludes establishing that such interventions improve outcomes. Future prospective studies are needed to evaluate whether personalized counseling improves adherence and maternal-infant health outcomes. This study is limited by its single-center, cross-sectional design and predominantly urban sample. Future research should employ multicenter designs with diverse populations to better understand factors associated with dietary intake and physical activity, thereby informing public health strategy development.

## Data Availability

The original contributions presented in this study are included in this article/supplementary material, further inquiries can be directed to the corresponding authors.
